# 3-Hydroxypropane-1,2-Diyl Dipalmitoleate—A Natural Compound with Dual Roles (CB1 Agonist/FAAH1 Blocker) in Inhibiting Ovarian Cancer Cell Line

**DOI:** 10.3390/ph14030255

**Published:** 2021-03-12

**Authors:** Christina Vijayaraghavan Sathynathan, Lakshmi Sundaram Raman, Sivamurugan Vajiravelu, Thirumal D. Kumar, Thyagarajan Sadras Panchatcharam, Gopinathan Narasimhan, George C. Priya Doss, Mary Elizabeth Gnanambal Krishnan

**Affiliations:** 1 Department of Biotechnology, Faculty of Biomedical Sciences and Technology, Sri Ramachandra Institute of Higher Education and Research (SRIHER), Deemed to be University (DU), Porur, Chennai, Tamil Nadu 600 116, India; christy.sathya@sriramachandra.edu.in; 2 Central Research Facility (CRF), Sri Ramachandra Institute of Higher Education and Research (SRIHER), Deemed to be University (DU), Porur, Chennai, Tamil Nadu 600 116, India; sundaram.79@gmail.com; 3 PG & Research Department of Chemistry, Pachaiyappa’s College, Chennai, Tamil Nadu 600 030, India; sivaatnus@gmail.com; 4 Department of Integrative Biology, School of Biosciences and Technology, Vellore Institute of Technology, Vellore, Tamil Nadu 632 014, India; thirumalkumar.d@gmail.com (T.D.K.); georgepriyadoss@vit.ac.in (G.C.P.D.); 5 Chancellor, Avinashilingam Institute for Home Science and Higher Education for Women (Deemed University), Coimbatore, Tamil Nadu 641 043, India; chancellor@avinuty.ac.in; 6 Department of Pharmaceutical Chemistry, Faculty of Pharmacy, Sri Ramachandra Institute of Higher Education and Research (SRIHER), Deemed to be University (DU), Porur, Chennai, Tamil Nadu 600 116, India; gopinathan.n@sriramachandra.edu.in

**Keywords:** Cannabinoid Receptor 1 (CB1), Fatty Acid Amide Hydrolase 1 (FAAH1), ovarian cancer, *Conus inscriptus*, 3-hydroxypropane-1,2-diyl dipalmitoleate, molecular modelling

## Abstract

Though it was once known that upregulated Cannabinoid Receptor (CB1) and downregulated Fatty Acid Amide Hydrolase (FAAH1) are associated with tumour aggressiveness and metastasis, it is now clear that upregulated CB1 levels more than a certain point cause accumulation of ceramide and directs cells to apoptosis. Hence, CB1 analogues/FAAH1 blockers are explored widely as anticancer drugs. There are reports on CB1-agonists and FAAH1-blockers separately, however, dual activities along with ovarian cancer-specific links are not established for any natural compound. With this setting, we describe for the first time the isolation of 3-hydroxypropane-1,2-diyl dipalmitoleate (564.48 Da) from a marine snail, *Conus inscriptus,* which binds to both CB1 and FAAH1 (glide energies: −70.61 and −30.52 kcal/mol, respectively). MD simulations indicate stable compound–target interaction for a minimum of 50 nanoseconds with relative invariabilities in *R_g_*. The compound inhibited ovarian cancer cell line, PA1 at 1.7 μM. Structural and chemical interpretation of the compound (**C2**) was done using FT-IR, GC-MS, ESI-MS, ^1^H and ^13^C-NMR (1 and 2D). Furthermore, a probable route for gram-scale synthesis of **C2** is hinted herein. With the available preliminary data, molecular mechanisms involving dual roles for this potent molecule must be elucidated to understand the possibilities of usage as an anticancer drug.

## 1. Introduction

It is well known that endocannabinoids (anandamide and 2-arachidonoyl glycerol) regulate varied physiological and neurological processes of a cell, including, memory [1], neurogenesis [2], appetite [3], homeostasis [4], and stress response [5]. These exciting molecules and their mimics partake in numerous pathways primarily by two receptors, Cannabinoid Receptor 1 and 2 (CB1 and CB2), besides some more putative targets. Of these, CB1 is primarily expressed in brain cells. Nevertheless, its expression is also reported in different locations including, spleen, heart, adrenal gland, ovaries, endometrium, and testes, among others [6,7]. Indeed, CB1 and their endogenous ligands are generally upregulated in the fast-growing cancer phenotypes to meet up their energy demands over the normal cell types. However, the irony is that when CB1 is expressed and stimulated more than a certain level, some surprising events like these happen: (i) accumulation of ceramide [8] and caspases causing an invariable release of proapoptotic factors and (ii) down-regulation of Matrix Metalloproteinases (MMPs), Vascular Endothelial Growth Factors (VEGF) of major types and survivins [9], which ultimately lead to cell death. Fatty Acid Amide Hydrolase (FAAH1), on the other hand, is a member of the serine hydrolase which is the principal catabolic enzyme of the endocannabinoid. Thus, CB1 agonists and FAAH1 blockers (to maintain the levels of agonist in the systemic circulation) together become plump targets in cancer therapies [10,11]. CB1 receptors, in general, show an increasing trend when treated with agonists in several cancer morphotypes; however, in normal tissue, these agonists decrease their expression. The differential expression may be a mechanism adopted by normal cells to get themselves protected from the pro-apoptotic effects of the routinely used CB1 agonists during cancer therapies [12].

It is known that any aberrant CB1 signalling could cause severe dysfunctioning of mitochondria [13,14] and it is also understood that there are several mitochondrial-mediated delicate apoptotic-regulating pathways in ovaries (menstrual cycle, granulocyte degeneration during folliculogenesis, corpus luteum regression, and many more) [15]. CB1 is therefore expressed in detectable levels in the reproductive and related organs, i.e., ovaries, anterior pituitary, and preoptic areas of the hypothalamus [16,17]. It is imperative to that FAAH1 is also expressed in tissues of the female reproductive system, including the ovaries, oviducts, endometrium, and myometrium [16]. Though many investigations are done in CB1 and FAAH1 as targets for varied cancer types [18,19,20], with regard to targeting ovarian cancer, there are only two reports: (i) localization and function of CB1 and FAAH1 in folliculogenesis, preovulatory follicle maturation, oocyte maturity and ovulation [6], and (ii) increased and differential expression of CB1 and FAAH1 in the ovaries at various stages from borderline to malignant tumours [21].

Gastropods are carnivorous invertebrates usually encountered in the neritic and deep ocean floors, which are known producers of more than 500,000 secondary metabolites [22,23], of which >90% are classified as peptides [23,24,25]. Voluminous investigations are available with aqueous extracts that contained the much-studied bioactive conopeptides which elicit neurological properties [26,27] and a few with anticancerous activities [28,29]. During the routine isolation of water-soluble molecules, also extracted are lipid-soluble compounds in quantities equal to/sometimes more than their aqueous counterparts. Anticancer related investigations or for that instance any other bioactivities are seldom studied for these lipophilic compounds. From our past experience, with a notion that fat solubles are also known exhibitors of bioactivities, we strictly followed the isolation protocols for obtaining the same, from the cone snail, *C. inscriptus,* and isolated six compounds, out of which, a hexane extractable 3-hydroxypropane-1,2-diyl dipalmitoleate is used for the current investigation. The molecule is active against human ovarian cancer cell line, PA1 at micromolar concentrations (1.7 μM) for which a range of assays was performed. For example, induction of apoptosis, cell migratory inhibitions, cell cycle arrest, shifts in Mitochondrial Membrane Potential (MMP), gene expression patterns during apoptosis were analyzed and a few more for which data is not shown here. Based on the in vitro results, we in this paper describe at length, isolation procedures of this compound along with structure elucidation details, toxicity analyses, ligand preparation, target prediction, integrated in silico modelling, molecular docking, and simulations using appropriate tools. Two main targets, Cannabinoid Receptor 1 (CB1) and Fatty Acid Amide Hydrolase 1 (FAAH1) (with more emphasis on the former), were identified for **C2**. CB1 has been a potential target to a few cancer types [18,30] with many controversies though. Alternatively, FAAH1 converts cannabinoid, an endogenous ligand for CB1 to arachidonic acid and play roles in fatty acid amide inactivation [31]. Hence, FAAHI blockers are also developed as one of the efficient cancer therapies so that CB1 signal transduction may happen uninterrupted to kill cancerous cells [19,32,33]. In addition, we also reviewed all plausible signal transduction pathways that could relate CB1 stimulation to cancer cell death. This paper attempts to provide an understanding of why **C2** could be used for inhibiting ovarian (for which preliminary data are provided herein) as well as other cancer types upon particularly binding to CB1. 

## 2. Results

### 2.1. Structural Investigations and Mass Spectral Analysis of C2 and Preliminary Bioactivities

An exhaustive investigation was made to elucidate the structure of the isolated compound based on the mass fragmentation patterns (Supplementary Figure S2) as seen in GC-MS, ESI-MS, and the combined details provided by ^1^H and ^13^C NMR spectral data. The predicted structure is provided as Supplementary Figure S3. A multiplet appearing at 5.35–5.40 ppm as seen in the ^1^H-NMR spectrum is due to the presence of alkenyl protons (-C**H**=C**H**-) attached to C_9_, C_10_, C_9′_ and C_10′_. The C_18_ carbon in glycerol moiety appearing at 4.63–4.73 ppm is shown as a multiplet, whereas a doublet at 4.38–4.45 ppm is due to the presence of C_19_ of glycerol methylene (-CH_2_-) proton attached to the oxygen atom of the fatty acid group to form an ester. A doublet at 3.80–3.83 ppm indicates the presence of C_17_ attached to glycerol -C**H_2_**OH. The multiplet appearing at 2.45–2.55 ppm in the ^1^H NMR spectrum is due to the presence of CH_2_ of C_2_ and C_2′_(α–CH_2_) next to a fatty acid carbonyl group (-OC(=O)**CH_2_**-CH_2_) followed by an alkyl chain. Whereas a multiplet at 1.95–2.05 ppm is due to the presence of allylic protons of C_8_, C_9_, C_8′_ and C_9′_(-C**H_2_**-CH=CH-C**H_2_**-) attached to a double bond. The vast multiplet appearing in the range 1.23 to 1.38 ppm is due to the presence of methylene (-CH_2_-) protons in the fatty acid alkyl chains. Terminal methyl (-CH_3_) protons of alkyl chains showed a multiplet at high field, 0.80–0.90 ppm. The isolated **C2** showed methane and methylene oxy protons, α-CH_2_ protons of fatty acid ester and the presence of more methylene and methyl protons profoundly revealed by relatively intense peaks showed the presence of a long fatty acid alkyl chain containing double bonds.

The presence of an ester carbonyl indicated at 171.5 ppm, and alkenyl carbons at 143.4 ppm in ^13^C NMR with relatively lesser intensities may be attributable to the number of carbon atoms. However, ^1^H NMR spectrum revealed the presence of –**CH_2_**C=O and alkenyl protons and allylic protons (CH = CH-C**H_2_**-), which also indicates that the carbon of α–CH_2,_ β-CH_2_ and γ-CH_2_ is attached to an ester carbonyl carbon showing signals at 37.1, 29.0 and 25.0 ppm, respectively. Huge multiplet signals appearing in the range of 39–40 ppm is attributable to DMSO-d_6_ solvent. Moreover, the methyl (-CH_3_) carbons of alkyl chains had signalled a singlet at 14.1 ppm, while the carbon of the allylic group (-**C**H_2_-**C**H_2_-CH=CH-**C**H_2_-**C**H_2_) appeared at 32.4, 29.7 and 29.4 ppm. Therefore, it could be concluded from ^13^C NMR spectroscopic details that the isolated sample may contain a glycerol ester of fatty acids with alkyl chains containing more than 16 carbons and a double bond. 

Whilst this with reference to NMR spectroscopic information, GC spectrum revealed two distinctive broad peaks, one at 27.63 and another at 28.33 min, and their mass fragmentation patterns coincided with the predicted structure. Apart from these two peaks, no other observable peaks appeared in the chromatogram, which could authenticate the purity of the sample. The purity of the sample was also confirmed by ESI-MS, and hence it was felt that GC-MS was sufficient to complete the structure elucidation of **C2** along with the support of NMR spectroscopy. The fragmentation pattern of two components obtained from GC indicates the presence of fatty acid of C_16_ containing a double bond in the alkyl chain. Furthermore, it confirms a signal at m/z at 237 that is indicative of the presence of palmitoleic acid acyl cation after losing –OH radical (m/z of palmitoleic acid is 253). Further, it was confirmed by another peak appearing at m/z 194, which is credited to the loss of acyl cation (-CH_2_CO)^+^. In addition, regular patterns of molecular fragments were observed at regular intervals by losing m/z 14 due to CH_2_ radicals, and the MS information linked to GC peak appearing at 28.32 min is discussed herewith. The spectrum follows the same fragmentation pattern as discussed above except a peak at m/z 317, which might have arisen from glycerol ester of palmitoleic acid with loss of water molecule from the glycerol unit. Observance of fragments at m/z 238 is due to the presence of palmitoleic acid acyl cation, and that of 208 is perhaps due to the elimination of carbonyl C = O from the acyl cation. Therefore, based on the spectral and chromatographic results from NMR and GC-MS fragmentation patterns, we conclude that **C2** is a bis(palmitoleic acid) ester of a glycerol with an IUPAC name: (9*Z*,9’*Z*)-3-hydroxypropane-1,2-diyl bis(hexadec-9-enoate) [3-hydroxypropane-1,2-diyl dipalmitoleate]; Mol. wt: 564.48 Da and all the specifics are given as Supplementary Figure S1. The chemical structure of the compound with all the spectral details were submitted publicly in the PubChem database, Substance Identification Number (SID): 404333224 [34] and Compound Identification Number (CID): 14275348 [35] were obtained.

The compound was titer tested to evaluate its efficiency to cause mortalities to PA1 ovarian cancer cell line and the IC_50_ value was calculated as 1.7 µM. AO/PI staining of cells revealed apoptotic bodies (Supplementary Figure S7B) and cell cycle analysis indicated **C2** treatment clearly piled up cells to G0/G1 phase. The concentration of **C2**, even if increased to 60 times than used for PA1 is not cytotoxic to non-cancerous Chinese Hamster Ovary (CHO) cell line, indicating the safety of the compound, which is also proved in ADMET test.

### 2.2. ADMET Analysis, Target Prediction, Preparation of Protein and Ligand Structures

**Figure 1:**
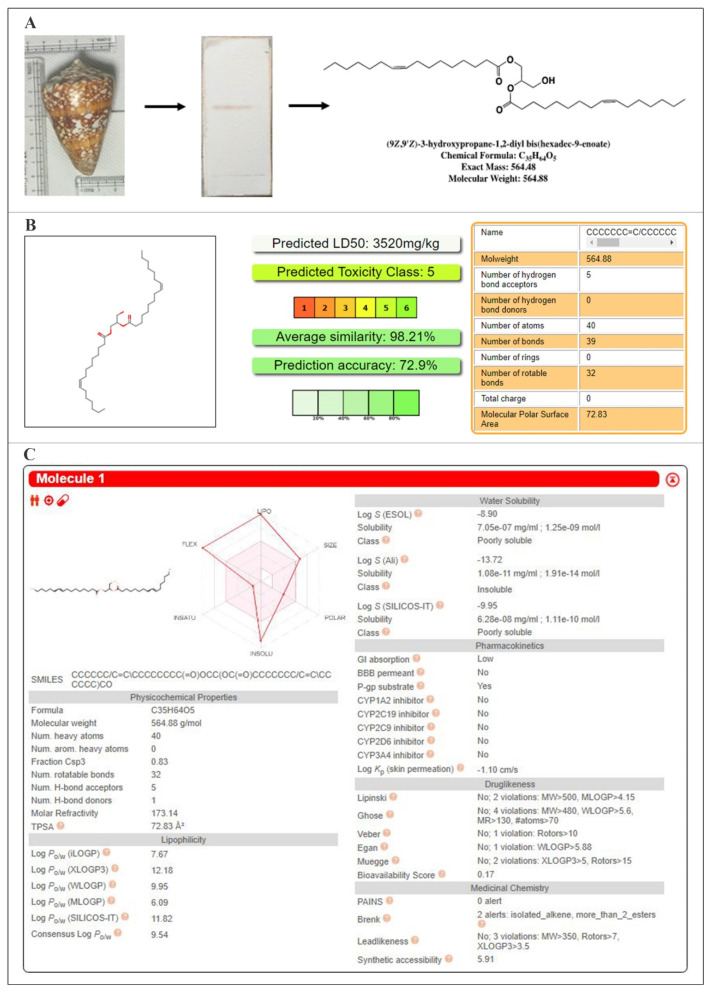
*Conus inscriptus* shell (left), Thin Layer Chromatogram (TLC) of the active compound (hexane: dichloromethane: 50:50 *v*/*v*; R*_f_*-0.5) (middle) and the structure of **C2** with molecular details (right) (**A**), Oral toxicity predictions—PROTOX (**B**) and physicochemical and pharmacokinetic properties—SwissADME (**C**).

Prediction of ADMET was made using the pkCSM tool [36], which evaluates the submitted compound for various pharmacokinetic properties (Supplementary Table S1). Physiochemical parameters such as lipophilicity, water-solubility, drug-likeness, and the medicinal chemistry aspects were evaluated using SwissADME [37] (Figure 1). **C2** compound showed lesser water solubility (−4.791); however, properties including human intestinal absorption, nil mutagenic, carcinogenic, hepatotoxic and AMES toxicities indicate the drug-likeness. Further, a toxicity prediction was made using ProTox-II server [38] and it was observed that **C2** showed the least toxicity and aligned under the Class 5 category.

**Figure 2:**
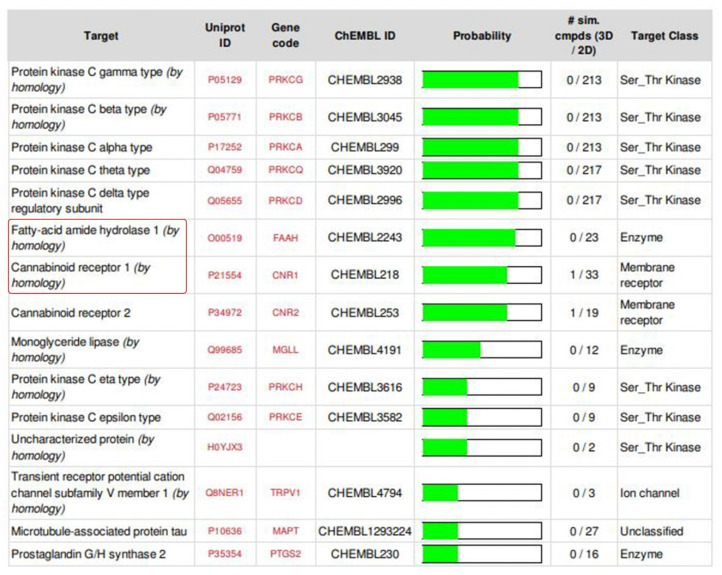
Select targets used for the present investigation (SwissTargetPrediction server (version 2014) http://old.swisstargetprediction.ch/ (accessed on 3 August 2021)).

Possible targets for the compound were predicted using SwissTargetPrediction server [39] with SMILES as input. Around 15 targets were listed as output, many of which were proteins (Figure 2)**,** out of which we chose Cannabinoid Receptor 1 (CB1) and the enzyme Fatty Acid Amide Hydrolase 1 (FAAH1) because both the targets work for Cannabinoid/Cannabidiol signaling. Another reason for leaving out the top listed targets is that these are very common targets and work for a huge network of transducing pathways, hence specificities may be doubtful. 

Three-dimensional structure is available for CB1 but not for FAAH1, hence, the latter was modelled using an online Swiss Model server which was evaluated using SAVES v5.0 server online (http://nihserver.mbi.ucla.edu/SAVES accessed on 3 August 2021). Calculations from the Ramachandran plot (PROCHECK v3.5) indicate that 89.5% of the residues were positioned in the favourable region, 10.1% in the allowed and only 0.3% were in the outlier region and the results are pictured as Supplementary Figure S4. Since less than 1% of residues were positioned in the outlier region, it can be opined that the model is satisfactory with considerable quality and authentication. PROSA is used to evaluate the quality of 3D models of protein structures. Z-score is a measure of overall model quality which denotes the deviation of the total energy of the structure compared to energy distribution derived from random conformations. It was found that the PROSA score was negative (−10.33) for the modelled protein, which indicates correctness. ERRAT is an “overall quality factor” for nonbonded atomic interactions and generally the scores directly proportionate to quality, with a generally accepted score of >50 for a high-quality model. For the current 3D model, the overall quality factor predicted by the ERRAT server was found to be 93.169 and Verify 3D server predicted that 94.18% of the residues in FAAH1 had an average 3D-1D score of ≥0.2, verifying the compatibility of the atomic model. The active sites of CB1 and FAAH1 were predicted using the SiteMap of Glide. 

### 2.3. Molecular Docking, Molecular Dynamics Simulations and Density Functional Theory (DFT) Calculations

**Figure 3:**
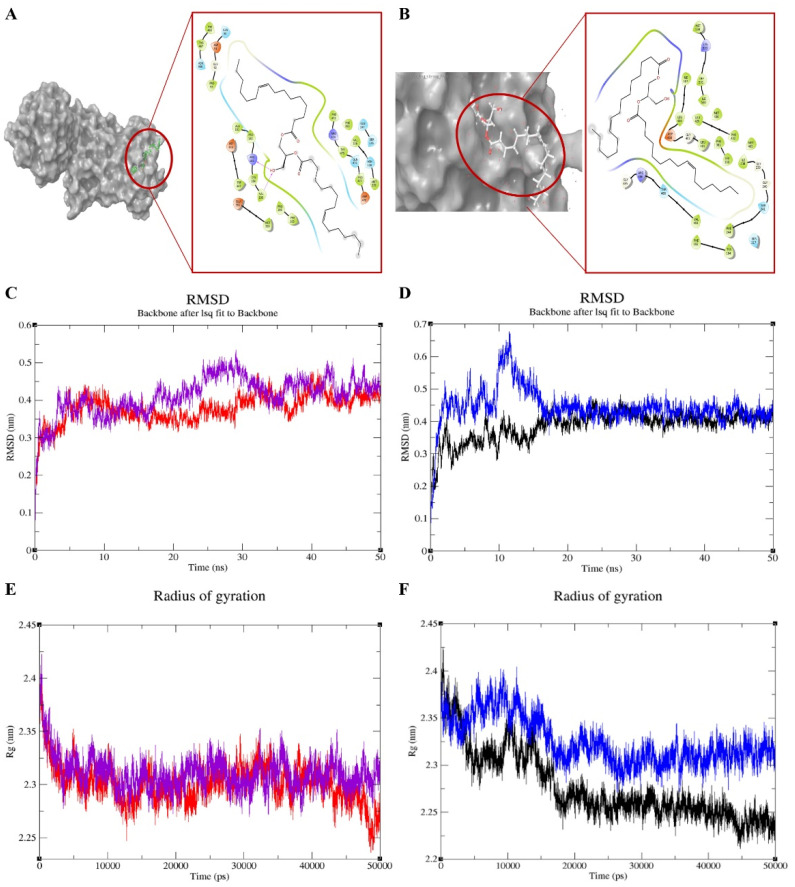
Molecular docking analysis (**A**,**B**), root mean square deviations (RMSD) (**C**,**D**), and radius of gyrations (**E**,**F**) for **C2**-target [cannabinoid receptor 1 (CB1) and fatty acid amide hydrolase (FAAH1)] complex [color coding: purple—CB1; red—CB1 with **C2**; blue—FAAH1; black—FAAH1 with **C2**].

Molecular docking scores and glide energies of CB1 and FAAH1 to **C2** were found to be −6.6 kcal/mol; −70.61 kcal/mol and −4.91 kcal/mol; −30.52 kcal/mol, respectively. CB1 was found to have formed two hydrogen bonds with **C2** at the VAL185 and ARG183 positions and numerous hydrophobic interactions at PHE473, ARG474, TRY475, ARG183, CYS184, VAL185, PRO106 and PHE105. FAAH1 was found to have only hydrophobic interactions at the positions, ARG486, THR488, LEU404, ILE407, GLY402, LEU401, PHE381, LEU 380, LEU429 and PHE432 (Supplementary Figure S5). Molecular dynamics simulations were performed to understand the dynamic behaviour of the protein–compound complex. Accordingly, the Root Mean Square Deviations (RMSD) of the molecules were analyzed to observe changes in the stability and convergence of the molecules over the simulation period. Results of molecular dynamics and simulation studies indicate that the targets (CB1 and/or FAAH1-bound forms) showed only minor fluctuations thus proving well-defined rotation of the targets, minimizing RMSD. Right from the start of the simulation run, CB1–**C2** complex showed little or no deviations at all, while that of FAAH1–compound-complex though showed slightly increased RMSD between 10 and 20 nanoseconds, however gained convergence and stability after 25 nanoseconds. Results of the RMSD analysis has given assurance to further analyze changes in compactness of these interacting proteins (Figure 3), by determining the Radius of Gyration (*R_g_*) in a GROMACS package. From the observed plots, it was known that CB1 and FAAH1-**C2** bound forms exhibited lesser *R_g_,* indicating tight packing and compactness. 

**Figure 4:**
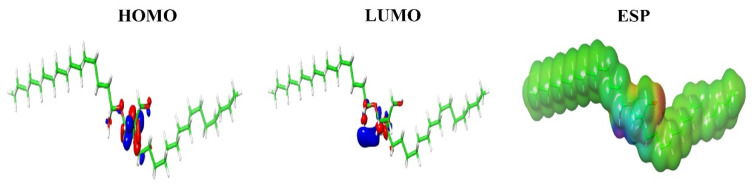
Prediction of highest occupied molecular orbital (HOMO), lowest unoccupied molecular orbital (LUMO) and electrostatic potential (ESP) regions of **C2**. The most negative (deep red) and positive electrostatic potential (deep blue) and the intermediate ranges of reactivity (orange, yellow, green) are shown here.

The possible occurrence of electrophilic and nucleophilic reactions within the molecule was predicted using Molecular Electrostatic Potential (MESP) and represented in Figure 4. As shown previously, the attractive and repulsive potentials are color-coded as red (negative) and blue (positive), respectively and the latter corresponds to the repulsion of proton by the atomic nuclei where there is a lesser electron cloud. However, there is no complete shielding of the nuclear charge and areas of orange, yellow and green indicated moderate charges. The main orbitals that take part in a chemical reaction are known as Highest Occupied Molecular Orbital (HOMO) and the least participants, Lowest Unoccupied Molecular Orbital (LUMO), and both these orbitals determine the charge transfers within the molecule. While HOMO is the outermost orbital containing electrons that could act as an electron donor, LUMO is the innermost orbital that acts as an electron acceptor. When the energy of HOMO is higher, the ability to donate electron is also higher and it directly relates to the ionization potential, whereas the energy of LUMO is directly related to the electron affinity. A transition state has been developed due to the interaction between the frontier orbitals (HOMO and LUMO) of **C2** to CB1. Table 1 lists the theoretical electrostatic parameters of the compound and it was noticed that HOMO-LUMO gap was lesser (−0.30917 eV), specifying chemical stability of the molecule. It was also noticed that the HOMO and LUMO are present over the Chlorine, Hydrogen, and Oxygen atoms towards the central part of **C2** and not in the fatty acid side chain, which may attribute to the lesser reactivity of the side chain as against the central part of the molecule (Figure 4). 

## 3. Discussion

In the current investigation, we isolated (9*Z*,9’*Z*)-3-hydroxypropane-1,2-diyl bis(hexadec-9-enoate), a 3-hydroxypropane-1,2-diyl dipalmitoleate that showed anticancerous properties in vitro, to ovarian cancer cells PA1 at 1.7 μM concentration. This compound is categorized under Class V, which indicates the least toxicity and harmful only if swallowed (2000 < LD50 ≤ 5000 mg/kg). Target prediction, in silico, indicates that **C2** binds to the Cannabinoid Receptor 1 (CB1) and Fatty Acid Amide Hydrolase 1 (FAAH1), whose common substrate is cannabinoid/cannabidiol. 

Though the output data from SwissTargetPrediction listed 15 targets, CB1 and FAAH1 were chosen for these reasons: (1) Protein Kinase C family (which appeared first in the list) are the commonest of the proteins investigated for cancer therapies and nothing new could probably be anticipated, and (2) both the chosen targets are interrelated in which one (FAAH1) modulates the availability of the ligand (cannabinoid/cannabidiol/**C2** in this case) and another governs CB1 pathway. In the present investigation, docking scores of −6.6 kcal/mol and −4.91 kcal/mol and the glide energies were observed as −70.61 kcal/mol and −30.52 kcal/mol, respectively, for CB1 and FAAH1 with the compound investigated. The glide energy for CB1 is almost twice lower (−70.61 kcal/mol) than that of FAAH1 (−30.52 kcal/mol) despite similar docking scores. It is generally shown that the ligands which form hydrophobic interactions with amino acids (in this case FAAH1) have higher glide scores. This can be a reason for lower glide energy for CB1, which forms hydrogen bonds with two amino acids. The type of bonds formed with amino acids by **C2** is clearly mentioned in Supplementary Figure S5.

The results indicate energetically favourable intermolecular interactions because of the negative ∆G values. When it comes to CB1, **C2** was found to have formed at least two hydrogen bonds and numerous hydrophobic bonds, whereas, FAAH1-compound complex established only hydrophobic interactions as mentioned in the results section. Since **C2** forms hydrogen bonds with CB1, it can be opined that minimal solubilities in water may be achieved when it is intended to be used as a drug in the future. Complete hydrophobic interaction of FAAH1 is in line with the fact that lipophilic drugs have a higher affinity for metabolizing enzymes. It is of the general opinion that the lipophilicity of a drug is proportionate to stronger binding affinities to proteins, distribution levels in systemic fluids, permeabilities and metabolic clearance [40,41]. With regard to RMSD studies, **C2** upon binding the targets showed some kind of deviation when the simulations were made up until 30 nanoseconds. However, there was a precise attainment of convergence after 30 nanoseconds and the pattern continued up to 50 nanoseconds. This could be taken as a reasonably good interaction for over a period of time long enough for the proceeding molecular pathways to get turned on. Changes in compactness of the proteins due to these interactions were measured using *R_g,_* and from the obtained plots we observed that the targets possessed reasonably invariant *R_g_* values throughout 50,000 picoseconds at default temperature 300 °K. These results assure the native conformation of the target proteins to maintain functionalities. Based on the DFT calculations, we conclude that the central part of the molecule might have interacted with CB1 receptor more than the side chain; however, the indirect participation of the latter cannot be ignored. In general, a smaller energy gap between LUMO and HOMO energies confers good bioactivity of a molecule. Because wider energy gap negatively affects the electron to move from the HOMO to the LUMO, which subsequently lead to a weak affinity between the ligand-target. The isolated molecule shows a smaller energy gap of “−0.30917”, which is attributable to the strong negative docking value and high ESP values as well.

**Figure 5:**
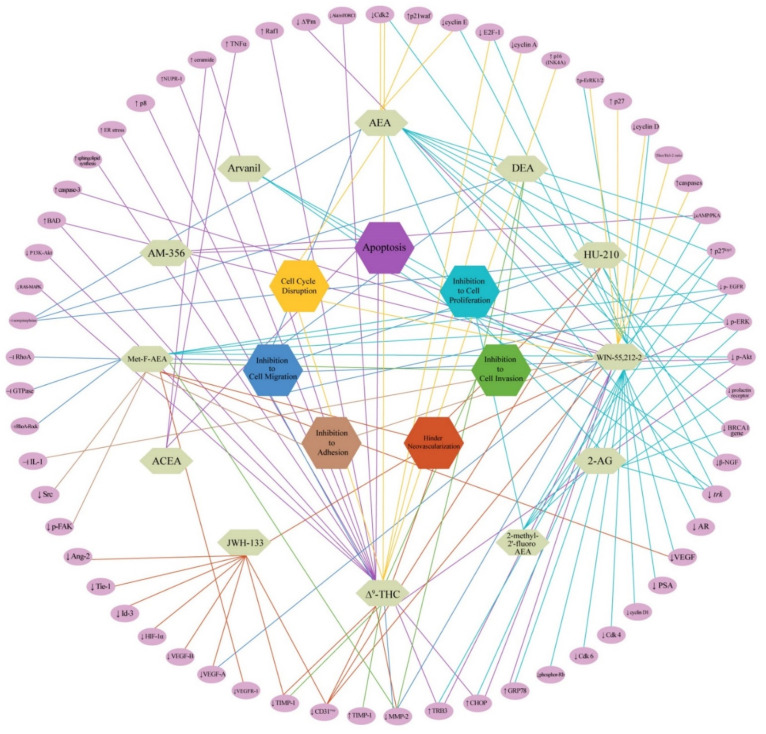
Molecular pathways-based target mapping for endocannabinoids and their mimics [Mode of cell death is completely reviewed and given in hexagonal boxes. Complete molecular target networking indicating expression levels of select proteins is provided as purple ovals in the periphery. Abbreviations: ↑—increased; ↓—decreased; ‒׀—inhibition; AEA—Anandamide; 2-AG—Arachidonoyl Glycerol; ACEA—Arachidonyl-2’-chloroethylamide; THC—Tetrahydrocannabinol; Met-F-AEA—2-Methyl-20-F-Anandamide; DEA—Docosatetraenylethanolamide; AM-35—-Methanandamide; Ang—Angiopoietin; ER—Endoplasmic reticulum; EGFR—Epidermal growth factor receptor; ERK—Extracellular signal-regulated kinase; Cdk—Cyclin-dependent kinase; FAK—Focal adhesion kinase; GRP—Glucose-regulated protein; MMP—Matrix metalloproteinase; TIMP—Tissue inhibitor matrix metalloproteinases; TRB—Tribblespseudokinase; VEGF—Vascular endothelial growth factor; VEGFR—Vascular endothelial growth factor receptor; PSA—Prostate-specific antigen; HIF—Hypoxia-inducible factor; IL—Interleukin; Tie-1—Angiopoietin receptor tyrosine kinase with immunoglobulin-like and epidermal growth factor-like domains 1; Id—Inhibitor of differentiation; AR—Androgen receptor; ERK—Extracellular regulating kinases; Akt—Protein kinase B; MAPK—Mitogen-activated protein kinase; RhoA-Rock—RhoA/Rho-associated coiled Coil-containing kinase; Raf—serine/threonine-protein kinase; NUPR—Nuclear protein.

Various mechanisms are postulated with respect to CB1 stimulation, with many controversies concerning the pathways/mechanisms/cell types/tumour progression, etc. Hence, we did an exhaustive pathway-based target mapping for all of the endocannabinoids/cannabidiols and their mimics highlighting the plausible mechanisms involved in cell death and reviewed almost all of the publications and a pictorial representation is made in Figure 5. The network exhibits polypharmacology of the existing cannabinoids and their analogues used in research till now, which is consistent with the broad spectrum of physiological responses that can be triggered. We indicate seven modes of cell death in cancerous cells when cannabinoid/cannabidiol analogues bind to CB1 and a range of molecular pathways (>60) and responses could be initiated. Till now, only 12 molecules (includes phytocannabinoids and their analogues) are being used in cancer therapies with different modes of action. Though a bird’s eye view of the target mapping indicates a complexity in the networking pathways, upon careful analysis one can understand that a single molecule has more often one than multiple ways to trigger cell death. This indicates that each molecule works by specific mechanisms with precise targets, which is the good news. It may be noted that inhibitions to cell proliferation and apoptosis are the most significant routes selected by the drug to kill the cells, whilst inhibitions to cell adhesion and invasions are the least chosen. However, it must also be understood that the targets for one cancer type may not be applicable to the other. For example, it is said that CB1 activation increases the levels of Tissue Inhibitor of MetalloProteinase 1 (TIMP1) in certain cancers to cause mortalities, whilst in glioblastoma multiforme, it actually was downregulated to produce the same effect [41].

Studies dealing with CB1 agonists usually parallel investigations on FAAH1 blockers as these ligands [whether endogenous or mimics] are metabolized by these enzymes. It is commonly known that the number of double bonds, length of the aliphatic chain, and the positions of the double bonds is directly proportional to enzyme inhibitory activities [42,43,44]. In the present case, **C2** has at least four double bonds which might have contributed to FAAH1 inhibitory activity. It can be noted that **C2** may thus not be taken for as a FAAH1 enhancer, in that case PA1 cell death at 1.7 μM concentration would not have been possible, owing to degradation or breakdown of **C2**, leading to a radical reduction in its concentration (given the strong affinities of enzyme-fatty acid substrates). 

A thorough literature search at the National Center for Biotechnology Information “PubChem” [45] was done and we understood that there are four other compounds, all with the same molecular weight, 564.9 Da, and chemical formula, C_35_H_64_O_5,_ like that of **C2** and the list is provided as Supplementary Table S2. Target prediction was also done for all of these compounds and it turned out to be the same targets (i.e., PKCδ, PKCθ, PKCα, PKCγ, CB1, CB2, Fatty acid Amidohydrolase/FAAH1) as those predicted for **C2**, however, with slight differences in the order of the occurrence of the targets in the list and the details are provided as Supplementary Figure S6. Out of these 4 compounds, no bioactivity is indicated for two, whereas an α-glucosidase inhibitory activity is reported for one (isolated from a sea cucumber) [44] and the last one is patented for usage as lipid bilayers, which could be applied to the surface of the nanopores with fluid walls for detection of biomolecules when they pass through. So, structurally related compounds are available and being added to the PubChem domain, which also have the same targets as **C2**. However, these compounds are not evaluated for anticancer properties, hitherto. So, this investigation discovers that compounds with structural similarities to **C2**, which apparently have similar targets could be explored both as CB1 and FAAH1 modulators for exploring anticancer activities.

**Scheme 1:**
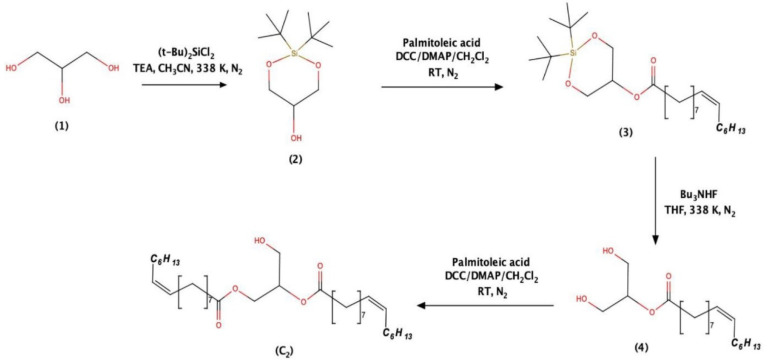
Synthesis of compound **C2.**

As a final part of the investigation, we propose a convenient synthetic route for the gram-scale production of **C2**. Selective protection of 1,3-hydroxyl group of glycerol (1) must be done using di-tert-butyldichlorosilane (DTBSCl_2_) catalysed by triethyl amine (TEA) under acetonitrile medium at 338 K. In this reaction, the formation of 1,3-protected glycerol (2) is more preferred than 1,2-protected glycerol. In the place of DTBSCl2, other silyl protecting reagents such as 1,3-dichloro-1,1,3,3-tetraisopropyldisiloxane (TIPDSCl_2_) and di-tert-butylsilyl bis(trifluoromethanesulfonate) (DTBS ditriflate) in DMF, can also be used. Further, the protected glycerol (2) is predicted to react with palmetoleic acid in the presence of DCC and DMAP in CH_2_Cl_2_, which would yield the intermediate (3). The di(t-butyl)silyl group in (3) cleaved using tert-butyl ammonium fluoride using THF as a solvent of choice could yield deprotected intermediate (4). Finally, by keeping 1:1 mole ratio of (4) and palmitoleic acid in the presence of DCC/DMAP at room temperature, **C2** yield can be anticipated in gram quantities (Scheme 1). The schema depicted in the synthesis sequence are high yielding reactions under mild conditions without the usage of heavy metals-based catalysts. The protection and deprotection reactions listed here are well-established reactions [46].

## 4. Materials and Methods

### 4.1. Processing of the Biological Material and Isolation of the Bioactive Compound

Fresh samples of *Conus inscriptus* collected at Tuticorin coastal areas (8.7642° N, 78.1348° E (120–130 m deep)) were brought to the laboratory and the soft bodies were taken off their shells. The material was washed thoroughly using double distilled water to remove debris and salt and air-dried thereafter. A sequential extraction procedure was followed and the samples were extracted with organic solvents from non-polar to high polarities in this order: hexane, dichloromethane, ethyl acetate and finally methanol. The samples were repeatedly extracted and the filtrates from each of these solvents were vacuum-distilled to dryness for assaying biological activities until the marc is no longer extractable. A Preparative Thin Layer Chromatography (*p*TLC) was performed to yield a brown coloured semisolid viscous compound (yield to dried snail biomass: 160 mg/500 g). This compound absorbs visible light but not UV (neither in long nor in short ranges). The pure compound had an R*_f_* value of 0.5 when hexane: dichloromethane (50:50 *v*/*v*) was used as eluent mixtures. The process for the isolation of **C2** is patented under Indian Jurisdiction. 

### 4.2. Structural Elucidation and Preliminary Bioassays of the Anticancer Molecule

The active compound was submitted to Fourier Transform-Infra Red (FT-IR) Spectroscopy (Jasco 4000 series, Maryland, USA) (5 mg of **C2** was compressed with 200 mg of KBr as pellets), Gas Chromatography-Mass Spectrometry (GC-MS) (Perkin Elmer, Clarus-600 GC/MS) [Column conditions: Phase Elite 35 MS [Capillary column (Agilent) dimensions: length—30.0 m; nominal diameter—250.00 μm; internal flow—1.4 mL/min; Column oven Temp: 50–280 °C; hold for 10 °C] and Electrospray Ionization-Mass Spectrometry (ESI-MS) (Shimadzu LCMS-8040 Triple Quadrupole System coupled with UHPLC (NEXERA) [MS- EI^+ve^ 70 eV; Iron source temperature: −200 °C, Column conditions: Flow rate: 350 µL min^−1^; DL Temp: 250 °C; Heat block: 450 °C]. For Nuclear Magnetic Resonance (NMR) spectroscopy, **C2** was dissolved in 0.6 mL of CDCl_3_ and fed to a Bruker Ultrashield 400 MHz (Avance III) machine for both ^1^H and ^13^C analyses [1 and 2D (COSY, DEPT-135, HMBC, and HSQC]. Tetramethylsilane (TMS) (0.03%) was used as an internal standard, and the chemical shifts of the protons signals were calculated from this. All the results were obtained and correlated to predict the structure of **C2**. The chemical and structural information of **C2** along with all the spectral details was submitted publicly in the PubChem database to obtain SID and CID. 

This compound possessed anticancer activities against a human teratocarcinoma ovarian cell line, PA1 with IC_50_ values of 1 µg mL^−1^ [3-(4,5-dimethylthiazol-2-yl)-2,5-diphenyltetrazolium bromide (MTT) assay [47]]. Microscopic images of Acridine Orange/Propidium Iodide (AO/PI) stained cells were taken in an Eclipse inverted microscope (Nikon T*i* E series, Japan, using fluorescent filters (CFI60). *Ex*/*Em*: AO-500/526 nm; PI-493/636 nm). **C2** was also tested in non-cancerous Chinese Hamster Ovary (CHO) to understand the safe usage of **C2** in normal cells. For cell cycle analysis, the cells were sorted in a MoFlo XDP Flow cytometer (Beckman Coulter, CA) at 488 nm (Supplementary Figure S7).

### 4.3. Bioinformatics: Tools and Methods

#### 4.3.1. ADMET Analysis, Target Prediction of the Isolated Compound and Structurally Similar Molecules, Preparation of Protein and Ligand Structures

Two-dimensional structure of the molecule was drawn and converted to a three-dimensional (3D) image using ChemSketch (v11.0) and tested for absorption, distribution, metabolism and excretion (ADMET) using SwissADME, pkCSM and ProTox-II servers. Plausible targets for **C2** were predicted using online SwissTargetPrediction server by providing the SMILES of the molecule as inputs both for ADMET and target prediction analysis. Protein sequences of CB1 and FAAH1 were retrieved from UniProt database with IDs P21554 and O00519, respectively. 3D structure of CB1 was retrieved from the Protein Data Bank with the PDB ID 5U09 [48] (resolution of 2.6 Å). The 3D structure of FAAH1 was modelled using online SWISS-MODEL [49] with template protein PDB ID 2WAP [50], which had an identity of 85.42% with the query sequence, and the model was evaluated using Structural Analysis and Verification Server (SAVES v5.0). When the chemical and structural details of **C2** were submitted in the PubChem as indicated earlier, we found that there are a few compounds at https://pubchem.ncbi.nlm.nih.gov (accessed on 3 August 2021) which were similar to **C2** and, therefore, target prediction was also done for all available compounds with structural similarities.

#### 4.3.2. Molecular Docking, Molecular Dynamics Simulations and Density Functional Theory (DFT) Calculations

Molecular docking analysis was performed using Glidemodule, 2018 of Schrödinger Suite (Schrödinger, LLC, New York, NY, 2018). Preparation of the ligand was done using LigPrep inbuilt in Schrödinger Suite, and the drawn ligand was geometrically-optimized by using Optimized Potentials for Liquid Simulations-2005 (OPLS-2005) force field which produced low-energy steric-free isomer of the ligand. Target proteins (CB1 and FAAH1) were prepared using the Protein Preparation Wizard of Schrödinger Suite. The tool has two gears: (i) preparation and (ii) refinement. The proteins were preprocessed separately by deleting the substrate cofactor as well as the crystallographically-observed water molecules (water without H bonds) after optimizing hydrogen bonds. After assigning charge and protonation state, energy minimization with Root Mean Square Deviation (RMSD = 0.30 Å fluctuation) was calculated by using the OPLS2005 force field. The prepared protein and the ligand were employed to build energy grids using the default value of protein atom scaling (1.0Å) within a cubic box centred on the centroid of the X-ray ligand pose. After grid generation, the ligand was docked with the protein using Glide-based ligand docking program of Schrödinger Suite in Extra Precision mode (XP), which used Monte Carlo Simulated Algorithm (MCSA)-based minimization. The best-docked pose showing the lowest Glide Score value was analyzed [51]. The binding energy was estimated using Prime Molecular Mechanics-Generalized Born Surface Area (MM-GBSA) module that comes with Schrödinger Suite 2018. The pose with the best docking score and binding energy was further used for visualization and molecular dynamics simulation process. Molecular dynamics simulations were performed using the GROMACS simulation package using the GROMOS96 53a6 force field [52]. The topology files of the small molecule were obtained using Automated Topology Builder (ATB) [53]. The system was neutralized using anti-charge ions (Na^+^ and Cl^−^). Further, energy minimization and equilibrations (NVT and NPT) were performed to attain system equilibrium. Finally, molecular dynamics simulations were performed for 50 nanoseconds. The trajectories were analyzed using g_rmsd and g_gyrate packages of GROMACS, and Grace software was used to plot the graphs. 

DFT analysis was done for the prepared molecule and all the computations were performed using Jaguar (v9.7) module of Schrödinger Suite (2018). All the following quantum chemical descriptors were computed: Molecular Electrostatic Potential (MESP), Highest Occupied Molecular Orbital (HOMO), Lowest Unoccupied Molecular Orbital (LUMO), and aqueous solvation energy. The entire accessible surface area of the molecule was sampled for electrostatic potentials at roughly the Van der Waals surface of the molecules and over the space extending beyond the molecular surface for the measurement of charge distribution. Areas in the molecule with positive electrostatic potential indicated excess positive charge and that of negative potential indicated regions of excess negative charge. The most negative was indicated as deep red, positive electrostatic potential in deep blue and areas of intermediate reactivities as orange, yellow and green [54]. Color-coded isosurface values provided an indication of the overall size of the molecule and the location of negative or positive electrostatic potentials. 

## 5. Conclusions

Based on all the previous reports on CB1 and FAAH1 ligands, we conclude that apart from being shown as a CB1 agonist, **C2** may also act as FAAH1 blocker which could have maintained its concentration in cancerous cells sufficient enough to stimulate CB1 pathway. Thus it may be speculated that this naturally occurring macromolecule in addition to being a CB1 agonist can also be a FAAH1 blocker simultaneously, in which case, dual roles must be appreciated. The anticancer compound, (9*Z*,9’*Z*)-3-hydroxypropane-1,2-diyl bis(hexadec-9-enoate) is active against PA1 cell line at 1.7 µM concentration, in vitro. Despite efficiency at a lower concentration, **C2** did not kill non-cancerous CHO cell line even when treated at 60 times more IC_50_ values than used for PA1. **C2** arrested cell cycle of PA1 precisely at G0/G1 phase indicating apoptotic route. in silico results, including lower binding energy, energetically favourable intermolecular interactions, and appreciable convergence with targets for a minimum of 50 nanoseconds indicate that this naturally occurring molecule could be explored as CB1 agonist-FAAH1 blocker. We propose that **C2**, which is easily extractable from *C. inscriptus* and its biosimilars reviewed in this paper could also be investigated for anticancer properties across a panel of cancer cells as CB1/FAAH1 modulators, so that these unique dual pathways can be explored.

## 6. Patents

The process for the isolation of **C2** is filed and published in Indian Patent Gazette with the following details: A process for extraction of bioactive compounds exhibiting anticancer property from *Conus inscriptus* and product thereof; Application No: E2/2723/2016/CHE.

## Figures and Tables

**Table 1:** Theoretical electrostatic parameters of the compound.

ID	HOMO (eV)	LUMO (eV)	HLG (eV)	ESP kCal/Mol
**C2**	−0.25527	0.05390	−0.30917	62.16

## Data Availability

All data are available upon request.

## Supplementary Materials

The following are available online at https://www.mdpi.com/1424-8247/14/3/255/s1, Figure S1. 1H (A), 13C (B) Nuclear Magnetic Resonance (NMR) Spectroscopy, Gas Chromatography-Mass Spectrum (GC-MS) datum (C), ESI-Mass (ESI-MS) spectrum (D) and Fourier-Transform Infrared (FT-IR) Spectroscopy (E); Figure S2. Possible fragmentation patterns (A and B) assigned to the compound in a Gas Chromatogram using a capillary column (5% Phenyl Methyl Siloxane); Figure S3. Predicted NMR structure of the compound; Figure S4. Modeled structure of FAAH1 (obtained on a SAVES v5.0 server) was evaluated for (A) energetically allowed regions in Ramachandran plot, (B) PROSA energy plot, (C) ERRAT2 score and (D) three-dimensional plot verification; Figure S5. Docked images of the compound with CB1 receptor and FAAH1 enzyme and the related datum; Figure S6. Prediction of targets for the chemically and structurally related compounds of 3-hydroxypropane-1,2-diyl dipalmitoleate using SwissTargetPredicition server (version 2019) (http://www.swisstargetprediction.ch/(accessed on 3 August 2021)); Figure S7. Inhibitory Concentration50 (IC50) values for the compound for PA1 cell line and non-cancerous CHO cell line and confirmatory data (MTT dropped plate) for assessing cell viabilities (The compound did not cause mortalities to non-cancerous cells even at IC50 value more than sixty folds that was used for PA1). Cell morphologies of A—PA1 untreated controls, B—PA1 treated with the compound, C—CHO untreated controls and D—CHO treated with the compound double-stained using Acridine Orange/ Propidium Iodide (AO/PI) and cell cycle analysis showing untreated PA1 controls with cells spanning all phases as against treated ones showing G0/G1 arrest; Table S1. ADMET analysis of the compound using pkCSM tool; Table S2. List of similar compounds from https://pubchem.ncbi.nlm.nih.gov (accessed on 3 August 2021) domain and their chemical and structural details.
