# Supplementary material for: 3-Hydroxypropane-1,2-Diyl Dipalmitoleate—A Natural Compound with Dual Roles (CB1 Agonist/FAAH1 Blocker) in Inhibiting Ovarian Cancer Cell Line
Source: Pharmaceuticals (Basel). 2021 Mar 12;14(3):255. doi: 10.3390/ph14030255 (PMC7998876; doi:10.3390/ph14030255)
Supplement: Supplementary file 1 [file pharmaceuticals-14-00255-s001.pdf]

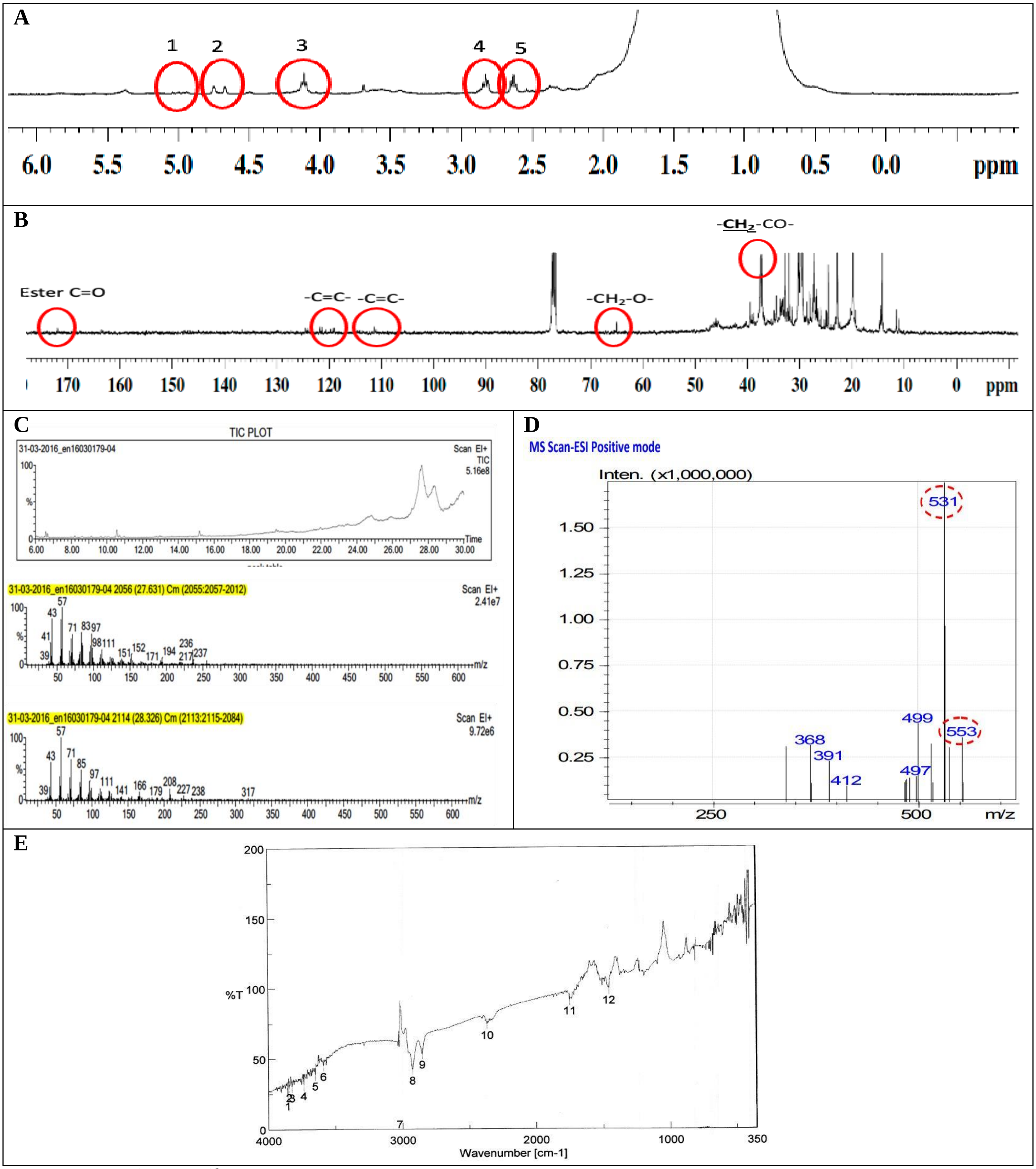

Figure S1. ^{1}H (**A**), ^{13}C (**B**) Nuclear Magnetic Resonance (NMR) Spectroscopy, Gas Chromatography-Mass Spectrum (GC-MS) datum (**C**), ESI-Mass (ESI-MS) spectrum (**D**) and Fourier-Transform Infrared (FT-IR) Spectroscopy (**E**)

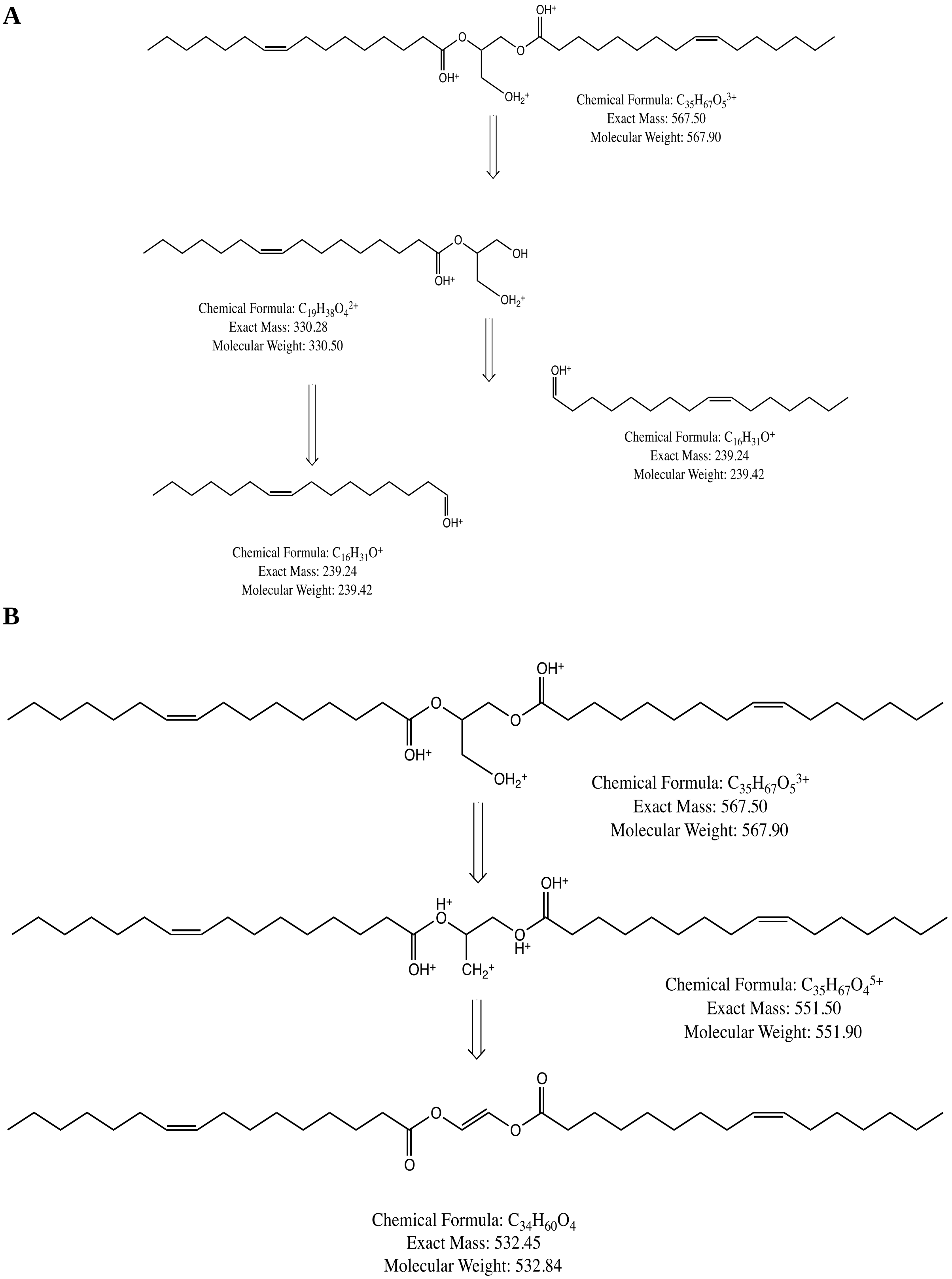

Figure S2. Possible fragmentation patterns (**A** and **B**) assigned to the compound in a Gas-Chromatogram using a capillary column (5% Phenyl Methyl Siloxane)

ChemNMR 1H Estimation

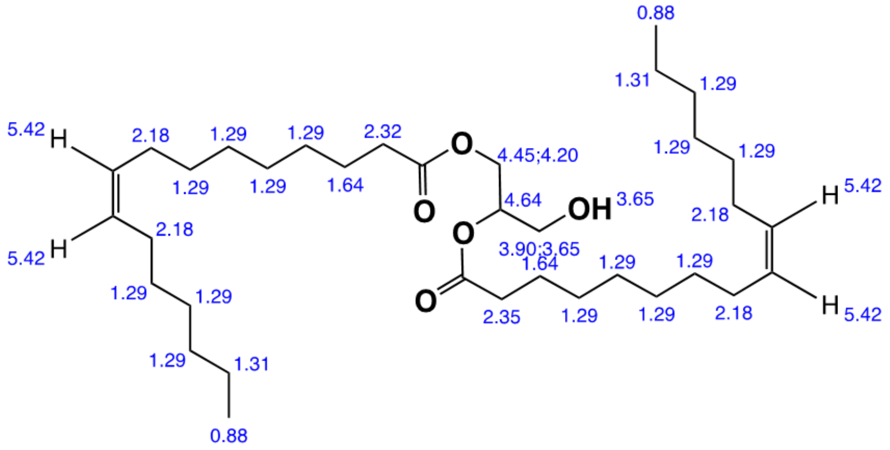

ChemNMR 13C Estimation

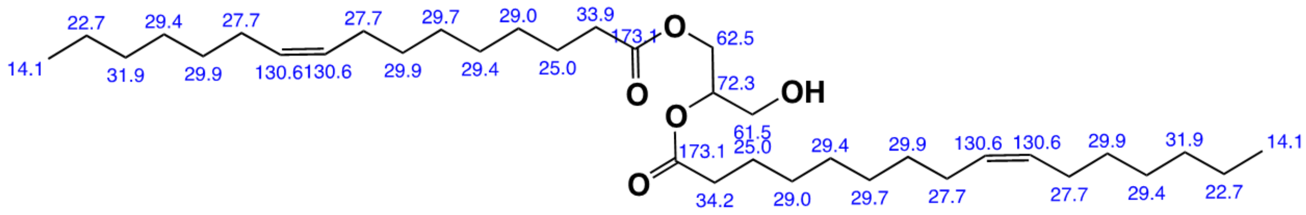

Figure S3. Predicted NMR structure of the compound

<u>Compound C2</u>: Yellow waxy solid; Yield: 0.0032% (160mg/ 500 dried biomass); ^{1}H NMR ($CDCl_3$, δ, ppm, 400 MHz): 5.05-5.10 (m, -CH=CH-, C_9 , C_9’, C_{10} and C_{10}’), 4.63-4.73 (m, >;CH-OCO-, C_{18}), 4.38-4.48 (d, -CH_2-O-, C_{19}), 3.80-3.83 (d, -CH_2-OH, C_{17}), 2.45-2.55 (t, -CH_2-(=O)O-, C_2 and C_2’), 1.95-2.05(m, -CH_2-CH=CH-, C_8 , C_8’, C_{11} and C_{11}’), 1.23-1.38 (m, -CH_2-), 0.80-0.90 (m,-CH_3); ^{13}C NMR($CDCl_3$, δ, ppm, 100 MHz): 171.5 (ester, -C(=O)O), 143.4 (-CH=CH-), 37.1 (-CH_2 -C(=O)O-),29.0 (-CH_2 -CH_2-COO), 25.0 (-CH_2-CH_2-H_2-COO), 32.4 (-CH_2-CH=CH-), 29.7 (-CH_2-CH_2-CH=CH-); GC-MS: GC (Rt, in): 7.63 m/z [M-OH] 236 (palmitoleic acid) Calcd: 254, GC(Rt, min): 28.33 m/z [M-OH] 317, [M-90] 227, [M-OH] 238 (palmitoleic acid).

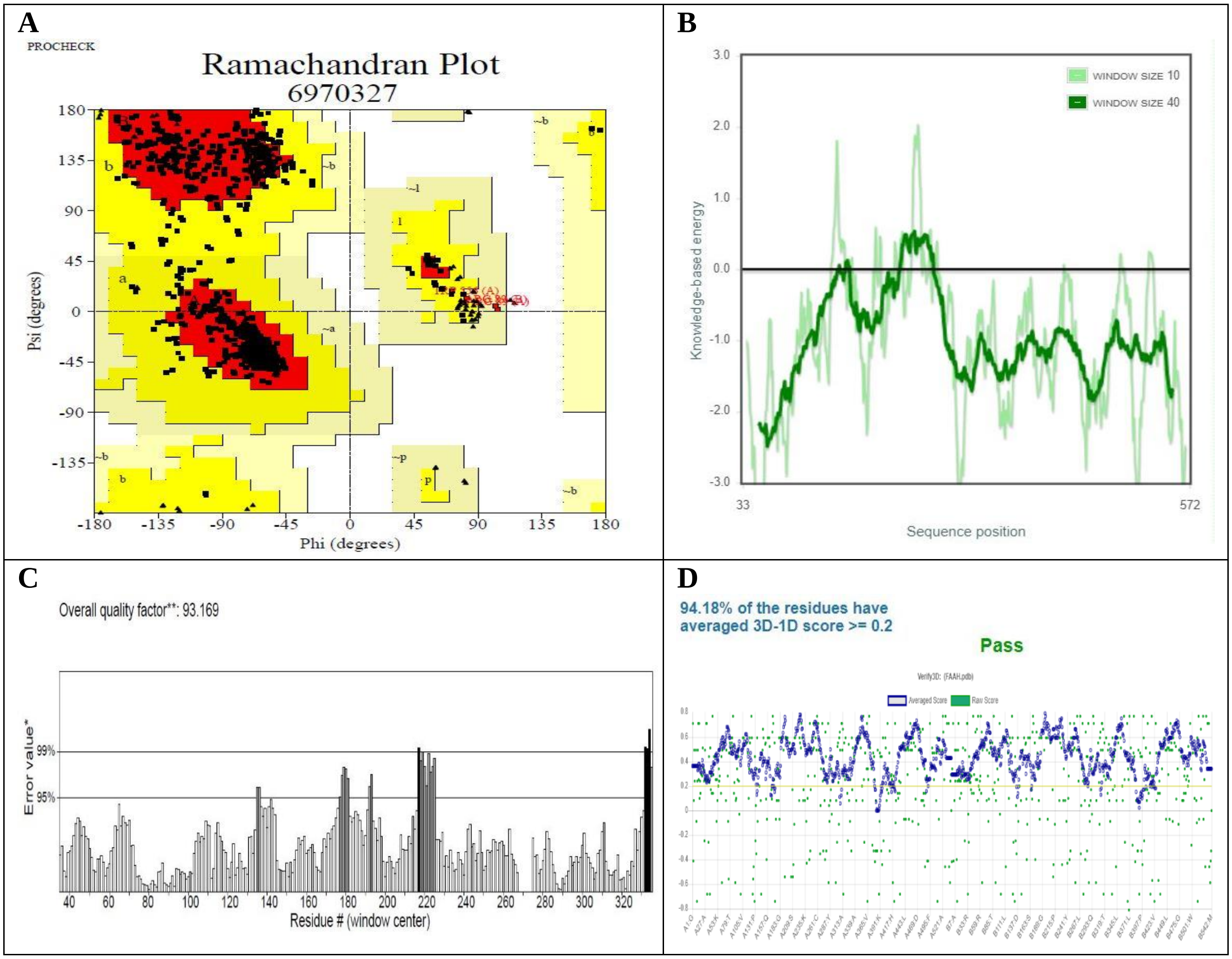

Figure S4. Modeled structure of FAAH1 (obtained on a SAVES v5.0 server) was evaluated for (**A**) energetically allowed regions in Ramachandran plot, (**B**) PROSA energy plot, (**C**) ERRAT2 score and (**D**) three-dimensional plot verification.

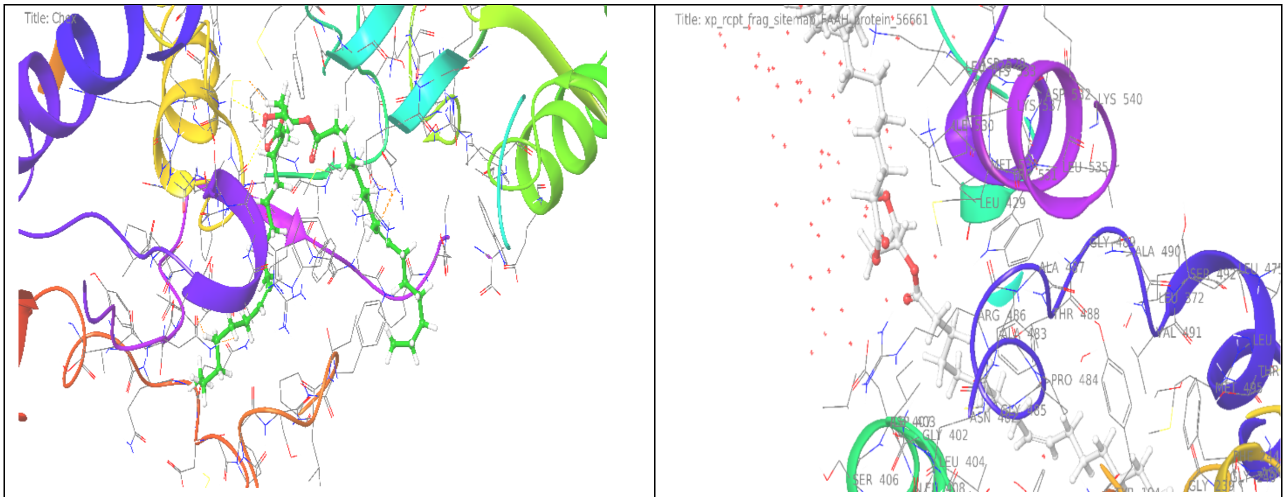

Macromolecule	Ligand	Docking Score	Binding Energy (kcal/mol)	Number of Hydrogen bonds formed	Hydrogen bond forming Amino acids	Hydrophobic interacting Amino acids
5U09 (CB1 protein)	Compound C2	-6.6	-70.61	2	VAL 185, ARG 183	PHE 473, ARG 474, TRY 475, ARG 183, CYS 184, VAL 185, PRO 106, PHE 105
FAAH1	Compound C2	-4.91	-30.52	-	-	ARG 486, THR 488, LEU 404, ILE 407, GLY 402, LEU 401, PHE 381, LEU 380, LEU 429, PHE 432

Figure S5. Docked images of the compound with CB1 receptor (left) and FAAH1 enzyme (right) (above) and the related datum (below)

Figure S6. Prediction of targets for the chemically and structurally related compounds of 3-hydroxypropane-1,2-diyl dipalmitoleate using Swiss Target Prediction server (version 2019) [http://www.swisstargetprediction.ch/]

1,2-di-(9Z-hexadecenoyl)-sn-glycerol

Target	Common name	Uniprot ID	ChEMBL ID	Target Class	Probability*	Known actives (3D/2D)
Protein kinase C delta	PRKCD	Q05655	CHEMBL2996	Kinase		1 / 32
Protein kinase C theta	PRKCQ	Q04759	CHEMBL3920	Kinase		1 / 8
Protein kinase C alpha	PRKCA	P17252	CHEMBL299	Kinase		1 / 227
11-beta-hydroxysteroid dehydrogenase 1	HSD11B1	P28845	CHEMBL4235	Enzyme		0 / 27
Anandamide amidohydrolase	FAAH	O00519	CHEMBL2243	Enzyme		0 / 9
Protein kinase C gamma *(by homology)*	PRKCG	P05129	CHEMBL2938	Kinase		0 / 4
Protein kinase C eta *(by homology)*	PRKCH	P24723	CHEMBL3616	Kinase		0 / 6
Cannabinoid receptor 1	CNR1	P21554	CHEMBL218	Family A G protein-coupled receptor		0 / 19
Cyclooxygenase-2	PTGS2	P35354	CHEMBL230	Oxidoreductase		0 / 15
Nitric oxide synthase, inducible *(by homology)*	NOS2	P35228	CHEMBL4481	Enzyme		0 / 14
Cannabinoid receptor 2	CNR2	P34972	CHEMBL253	Family A G protein-coupled receptor		1 / 6
HMG-CoA reductase *(by homology)*	HMGCR	P04035	CHEMBL402	Oxidoreductase		0 / 99
T-cell protein-tyrosine phosphatase	PTPN2	P17706	CHEMBL3807	Phosphatase		0 / 26
Autotaxin	ENPP2	Q13822	CHEMBL3691	Enzyme		0 / 25
Lysophosphatidic acid receptor 6	LPAR6	P43657	CHEMBL2331058	Family A G protein-coupled receptor		0 / 6

2,3-dipalmitoleoyl-sn-glycerol

Target	Common name	Uniprot ID	ChEMBL ID	Target Class	Probability*	Known actives (3D/2D)
Protein kinase C delta	PRKCD	Q05655	CHEMBL2996	Kinase		1 / 32
Protein kinase C theta	PRKCQ	Q04759	CHEMBL3920	Kinase		1 / 8
Protein kinase C alpha	PRKCA	P17252	CHEMBL299	Kinase		1 / 227
11-beta-hydroxysteroid dehydrogenase 1	HSD11B1	P28845	CHEMBL4235	Enzyme		0 / 27
Anandamide amidohydrolase	FAAH	O00519	CHEMBL2243	Enzyme		0 / 9
Protein kinase C gamma *(by homology)*	PRKCG	P05129	CHEMBL2938	Kinase		0 / 4
Protein kinase C eta *(by homology)*	PRKCH	P24723	CHEMBL3616	Kinase		0 / 6
Cannabinoid receptor 1	CNR1	P21554	CHEMBL218	Family A G protein-coupled receptor		0 / 19
Cyclooxygenase-2	PTGS2	P35354	CHEMBL230	Oxidoreductase		0 / 15
Nitric oxide synthase, inducible *(by homology)*	NOS2	P35228	CHEMBL4481	Enzyme		0 / 14
Cannabinoid receptor 2	CNR2	P34972	CHEMBL253	Family A G protein-coupled receptor		1 / 6
HMG-CoA reductase *(by homology)*	HMGCR	P04035	CHEMBL402	Oxidoreductase		0 / 99
T-cell protein-tyrosine phosphatase	PTPN2	P17706	CHEMBL3807	Phosphatase		0 / 26
Autotaxin	ENPP2	Q13822	CHEMBL3691	Enzyme		0 / 25
Lysophosphatidic acid receptor 6	LPAR6	P43657	CHEMBL2331058	Family A G protein-coupled receptor		0 / 6

1,3-dipalmitolein

Target	Common name	Uniprot ID	ChEMBL ID	Target Class	Probability*	Known actives (3D/2D)
Protein kinase C delta	PRKCD	Q05655	CHEMBL2996	Kinase		0 / 35
Protein kinase C alpha	PRKCA	P17252	CHEMBL299	Kinase		0 / 223
Protein kinase C theta	PRKCQ	Q04759	CHEMBL3920	Kinase		0 / 10
11-beta-hydroxysteroid dehydrogenase 1	HSD11B1	P28845	CHEMBL4235	Enzyme		0 / 30
Anandamide amidohydrolase	FAAH	O00519	CHEMBL2243	Enzyme		0 / 11
Prostaglandin E synthase	PTGES	O14684	CHEMBL5658	Enzyme		0 / 13
Cyclooxygenase-2	PTGS2	P35354	CHEMBL230	Oxidoreductase		0 / 15
Cannabinoid receptor 1 *(by homology)*	CNR1	P21554	CHEMBL218	Family A G protein-coupled receptor		0 / 27
Cannabinoid receptor 2 *(by homology)*	CNR2	P34972	CHEMBL253	Family A G protein-coupled receptor		0 / 7
HMG-CoA reductase *(by homology)*	HMGCR	P04035	CHEMBL402	Oxidoreductase		0 / 99
Protein-tyrosine phosphatase 1B	PTPN1	P18031	CHEMBL335	Phosphatase		0 / 81
Lysophosphatidic acid receptor 6	LPAR6	P43657	CHEMBL2331058	Family A G protein-coupled receptor		0 / 5
Lysophosphatidic acid receptor Edg-7	LPAR3	Q9UBY5	CHEMBL3250	Family A G protein-coupled receptor		0 / 25
Autotaxin	ENPP2	Q13822	CHEMBL3691	Enzyme		0 / 25
Lysophosphatidic acid receptor Edg-4	LPAR2	Q9HBW0	CHEMBL3724	Family A G protein-coupled receptor		0 / 17

1-linoleoyl-2-myristoyl-sn-glycerol

Target	Common name	Uniprot ID	ChEMBL ID	Target Class	Probability*	Known actives (3D/2D)
Protein kinase C theta	PRKCQ	Q04759	CHEMBL3920	Kinase		0 / 8
Protein kinase C alpha	PRKCA	P17252	CHEMBL299	Kinase		1 / 212
Protein kinase C delta	PRKCD	Q05655	CHEMBL2996	Kinase		1 / 31
Cannabinoid receptor 1	CNR1	P21554	CHEMBL218	Family A G protein-coupled receptor		22 / 21
Cannabinoid receptor 2	CNR2	P34972	CHEMBL253	Family A G protein-coupled receptor		15 / 8
Anandamide amidohydrolase	FAAH	O00519	CHEMBL2243	Enzyme		1 / 9
11-beta-hydroxysteroid dehydrogenase 1	HSD11B1	P28845	CHEMBL4235	Enzyme		0 / 24
Protein kinase C gamma *(by homology)*	PRKCG	P05129	CHEMBL2938	Kinase		0 / 4
Protein kinase C epsilon	PRKCE	Q02156	CHEMBL3582	Kinase		0 / 28
Protein kinase C eta *(by homology)*	PRKCH	P24723	CHEMBL3616	Kinase		0 / 6
Cyclooxygenase-2	PTGS2	P35354	CHEMBL230	Oxidoreductase		0 / 15
Nitric oxide synthase, inducible *(by homology)*	NOS2	P35228	CHEMBL4481	Enzyme		0 / 11
Methionine aminopeptidase 2	METAP2	P50579	CHEMBL3922	Protease		0 / 9
Cytochrome P450 19A1	CYP19A1	P11511	CHEMBL1978	Cytochrome P450		0 / 58
HMG-CoA reductase *(by homology)*	HMGCR	P04035	CHEMBL402	Oxidoreductase		0 / 92

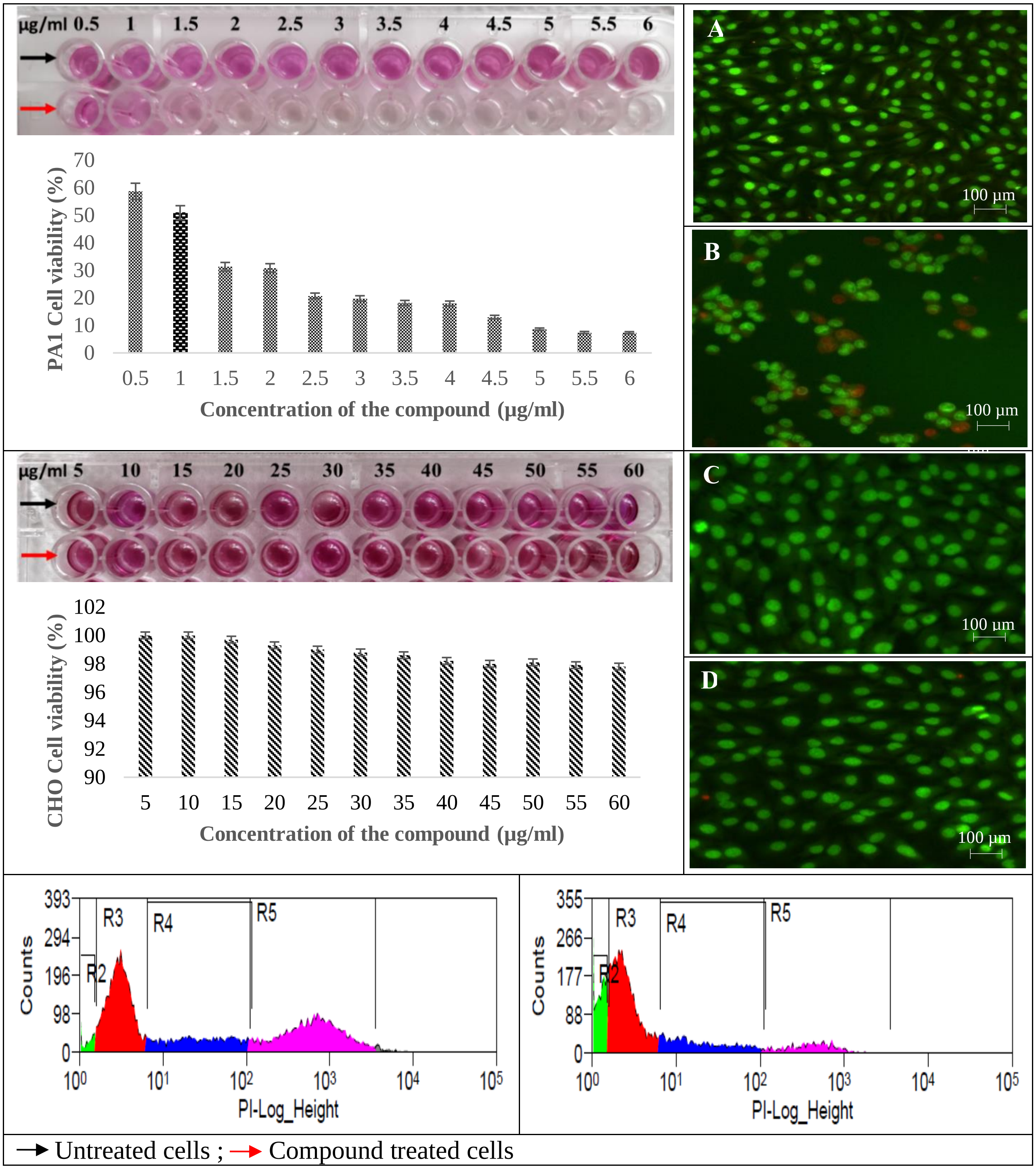

Figure S7. Inhibitory Concentration$_{50}$ (IC_{50}) values for the compound for PA1 cell lines and non-cancerous CHO cell lines and confirmatory data [MTT dropped plates] for assessing cell viabilities (above the corresponding graphs) [The compound did not cause mortalities to non-cancerous cells even at IC_{50} value more than sixty folds that was used for PA1]. Cell morphologies of **A**- PA1 untreated controls; **B**- PA1 treated with the compound; **C**- CHO untreated controls and **D**- CHO treated with the compound double-stained using Acridine Orange/ Propidium Iodide [AO/PI] and cell cycle analysis showing untreated PA1 controls (left far below) with cells spanning all phases as against treated ones showing G0/G1 arrest (right far below)

Table S1. ADMET analysis of the compound using pkCSM tool

ADMET prediction using pkCSM tool		
**Property**	**Parameters**	**Values**
Absorption	Water solubility	-4.791
Absorption	Caco2 permeability	0.023
Absorption	Intestinal absorption (human)	86.819
Absorption	Skin Permeability	-2.734
Absorption	P-glycoprotein substrate	No
Absorption	P-glycoprotein I inhibitor	No
Absorption	P-glycoprotein II inhibitor	Yes
Distribution	VDss (human)	-0.887
Distribution	Fraction unbound (human)	0.09
Distribution	BBB permeability	-0.872
Distribution	CNS permeability	-3.009
Metabolism	CYP2D6 substrate	No
Metabolism	CYP3A4 substrate	Yes
Metabolism	CYP1A2 inhibitor	No
Metabolism	CYP2C19 inhibitor	No
Metabolism	CYP2C9 inhibitor	No
Metabolism	CYP2D6 inhibitor	No
Metabolism	CYP3A4 inhibitor	No
Excretion	Total Clearance	2.215
Excretion	Renal OCT2 substrate	No
Toxicity	AMES toxicity	No
Toxicity	Max. tolerated dose (human)	0.117
Toxicity	hERG I inhibitor	No
Toxicity	hERG II inhibitor	No
Toxicity	Oral Rat Acute Toxicity (LD50)	2.062
Toxicity	Oral Rat Chronic Toxicity (LOAEL)	0.557
Toxicity	Hepatotoxicity	No
Toxicity	Skin Sensitisation	No
Toxicity	*T.pyriformis* toxicity	0.286
Toxicity	Minnow toxicity	-6.007

Table S2. List of similar compounds from https://pubchem.ncbi.nlm.nih.gov domain and their chemical and structural details

Name	Chemical Formula	Molecular Weight	IUPAC Name	Structure
Isolated Compound **C2**<br>PubChem CID: 14275348	$C_{35}H_{64}O_5$	564.88	(9*Z*,9'*Z*)-3-hydroxypropane-1,2-diyl bis(hexadec-9-enoate)	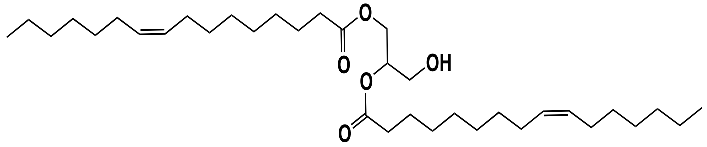
1,2-Di-(9Z-hexadecenoyl)-sn-glycerol<br><br>PubChem CID: 9543679	$C_{35}H_{64}O_5$	564.9	[(2*S*)-2-[(*Z*)-hexadec-9-enoyl]oxy-3-hydroxypropyl] (*Z*)-hexadec-9-enoate	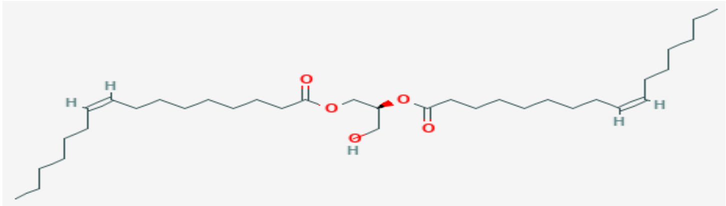
2,3-Dipalmitoleoyl-sn-glycerol<br><br>PubChem CID: 10325582	$C_{35}H_{64}O_5$	564.9	[(2*R*)-2-[(*Z*)-hexadec-9-enoyl]oxy-3-hydroxypropyl] (*Z*)-hexadec-9-enoate	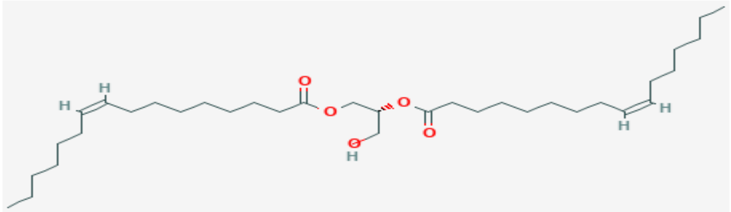
1,3-Dipalmitolein<br><br>PubChem CID: 45934047	$C_{35}H_{64}O_5$	564.9	[3-[(*Z*)-hexadec-9-enoyl]oxy-2-hydroxypropyl] (*Z*)-hexadec-9-enoate	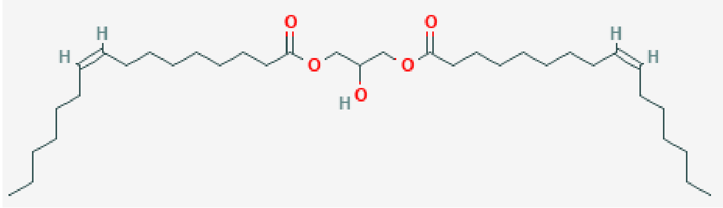
1-Linoleoyl-2-myristoyl-sn-glycerol<br><br>PubChem CID: 53478098	$C_{35}H_{64}O_5$	564.9	[(2*S*)-3-hydroxy-2-tetradecanoyloxypropyl] (9*Z*,12*Z*)-octadeca-9,12-dienoate	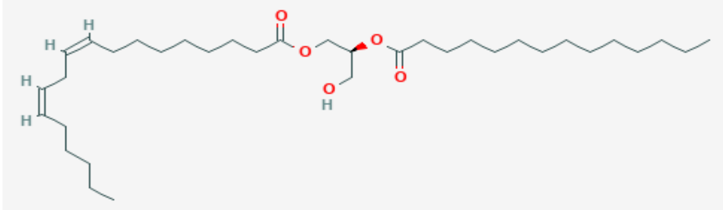
